# Testing the inhibitory cascade model in Mesozoic and Cenozoic mammaliaforms

**DOI:** 10.1186/1471-2148-13-79

**Published:** 2013-04-08

**Authors:** Thomas JD Halliday, Anjali Goswami

**Affiliations:** 1 Department of Earth Sciences, University College London, Gower Street, London, UK; 2 Department of Genetics, Evolution and Environment, University College London, Gower Street, London, UK

**Keywords:** Mammalia, Developmental constraints, Teeth, Fossils

## Abstract

**Background:**

Much of the current research in the growing field of evolutionary development concerns relating developmental pathways to large-scale patterns of morphological evolution, with developmental constraints on variation, and hence diversity, a field of particular interest. Tooth morphology offers an excellent model system for such ‘evo-devo’ studies, because teeth are well preserved in the fossil record, and are commonly used in phylogenetic analyses and as ecological proxies. Moreover, tooth development is relatively well studied, and has provided several testable hypotheses of developmental influences on macroevolutionary patterns. The recently-described Inhibitory Cascade (IC) Model provides just such a hypothesis for mammalian lower molar evolution. Derived from experimental data, the IC Model suggests that a balance between mesenchymal activators and molar-derived inhibitors determines the size of the immediately posterior molar, predicting firstly that molars either decrease in size along the tooth row, or increase in size, or are all of equal size, and secondly that the second lower molar should occupy one third of lower molar area. Here, we tested the IC Model in a large selection of taxa from diverse extant and fossil mammalian groups, ranging from the Middle Jurassic (~176 to 161 Ma) to the Recent.

**Results:**

Results show that most taxa (~65%) fell within the predicted areas of the Inhibitory Cascade Model. However, members of several extinct groups fell into the regions where m2 was largest, or rarely, smallest, including the majority of the polyphyletic “condylarths”. Most Mesozoic mammals fell near the centre of the space with equality of size in all three molars. The distribution of taxa was significantly clustered by diet and by phylogenetic group.

**Conclusions:**

Overall, the IC Model was supported as a plesiomorphic developmental system for Mammalia, suggesting that mammal tooth size has been subjected to this developmental constraint at least since the divergence of australosphenidans and boreosphenidans approximately 180 Ma. Although exceptions exist, including many ‘condylarths’, these are most likely to be secondarily derived states, rather than alternative ancestral developmental models for Mammalia.

## Background

### Inhibitory cascade model

Tooth morphology is used extensively in the study of mammalian evolution because teeth are generally well-preserved in the fossil record and contain a large amount of phylogenetically and ecologically important information [1]. With the explosion of the field of ‘evo-devo’ over the last few decades [2], new data on tooth development have provided broad hypotheses on the mechanisms generating the diversity of morphologies observed in mammalian teeth (e.g. [3,4]). These hypotheses have, however, rarely been applied to palaeontological datasets, due to the difficulty of discerning developmental mechanisms in the fossil record (but see [5] and references therein).

Across mammals, molar buds develop sequentially from the anteriormost to the posteriormost [6,7]. In a recent study, Kavanagh *et al*. [8] examined lower molar development in extant murid rodents, demonstrating that explantation of lower molar buds delayed development of posterior molars, but that early severance of posterior molar buds restored the rate of growth. In the framework of their model, termed an ‘Inhibitory Cascade’ (IC), the growth of each developing molar bud is affected by the balance between an inhibitor present in the adjoining anterior molar and a mesenchymal activator. A key feature of the IC Model is that the changes in size along the molar sequence will be cumulative – in other words, the development of the third lower molar (m3) is affected both by m2 and m1. The parameters of this cumulative relationship, determined experimentally, predict that, should the IC Model be a primary control on mammalian tooth sizes, m2 will occupy one third of total molar occlusal area, regardless of whether m1 is larger than m3 or vice versa. The second lower molar, then, will always be intermediate in size, or all three molars will be the same size. They further demonstrated that this pattern was broadly applicable across murid rodents. A third prediction suggested is that there is a correlation between the position of a taxon in the molar morphospace and its diet. Specifically, they state that “the most equal molar proportions are found in herbivorous taxa and the least equal in faunivorous taxa”, and demonstrate this prediction with one example each of a faunivorous, omnivorous, and herbivorous murid, although this is not tested statistically across murids.

As a developmental mechanism, the IC Model is unusual in providing testable predictions regarding morphologies which are readily preserved in the fossil record. This applicability to taxa that are only available as fossilised remains and hence generally excluded from such analyses allows for robust testing of the origin of the mechanism itself. Teeth are among the best preserved elements of a mammalian skeleton and make up a significant proportion of specimens found in mammalian assemblages (e.g. [9]). For this reason, many extinct taxa that are known solely from a lower molar series can be included in an analysis of the IC Model, thus greatly increasing the potential dataset available for study.

A small number of studies have tested the predictions of the IC Model in a variety of fossil and extant mammalian groups [10,11]. The predictions of the IC Model have been found to, for the most part, be applicable to Rodentia [12] as a whole, and South American ungulates [13], although in each case, several taxa fell outside of the expected area. Thus far, the largest deviation from the predictions of the IC Model has been found in canids (dogs and their kin) [11], but also in arvicoline rodents (voles and lemmings) [14], leading the latter authors to conclude that the IC Model might not be generalisable even across rodents. In contrast, an analysis of 29 mammals, mostly extant placental mammals but also including two marsupials and some extinct taxa, suggests that the IC Model held true for all variation across the sample, although there were some outliers [10]. The distribution of taxa in that study also supported the prediction that taxa with different diets would fall into distinct regions of the molar morphospace, with herbivorous forms bearing relatively larger m3, and faunivorous relatively larger m1, although this was again not tested statistically. That the IC Model has been supported in detailed analysis of two disparate groups (the South American notoungulates and most rodents), as well as a phylogenetically broad sample of predominantly extant mammals, suggests that this developmental mechanism may have been established early in mammalian evolution.

In this study, we test the applicability of the IC Model within a large sample of extant and extinct boreosphenidans (the clade including extant marsupials, placentals, and their stem relatives) and australosphenidans (the clade including monotremes and their stem relatives). These lineages are estimated to have diverged approximately 180 million years ago (Ma) and encompass all of extant mammalian diversity [15,16]. Thus, the sample has sufficient phylogenetic breadth to assess the hypothesis that the IC Model is a common and ancestral model of mammalian tooth development and that it was established early in mammalian evolution.

## Methods

### Taxonomic sampling

A total of 154 specimens were included in the present study (Additional file 1), comprising 132 genera within 23 orders. The majority of these taxa are eutherians, including placental mammals and their stem relatives. Within placental mammals, the four superorders were all sampled. The “southern” superorders Xenarthra (sloths, armadillos and anteaters) and Afrotheria (elephants, hyraxes, sirenians and allies) were each represented by two genera. Euarchontoglires was represented by two scandentians (tree shrews), two dermopterans (colugos), three primates and ten rodents. The best sampled of the four superorders was Laurasiatheria, with three sampled from Carnivora (cats, dogs, bears and allies), ten from Perissodactyla (horses, rhinoceroses, and tapirs), fifteen from Eulipotyphla (shrews, moles and allies), and seventeen from Artiodactyla (cows, pigs, camels and allies).

In addition to those taxa known to fit unambiguously within extant placental orders, several stem taxa and taxa of uncertain affinities were included. Among those sampled taxa of less certain affinities are three genera of Arctostylopidae, a group which has traditionally been placed with the notoungulates (e.g. [17]), but which more recent studies place near the stem of Glires (rodents, rabbits and pikas) [18]. Notoungulates (two representatives) is one of several South American ungulate clades [19] generally treated as Mammalia *incertae sedis*[20], although they have been reconstructed as close to Afrotheria based on shared dental, vertebral and astragalar synapomorphies [21]. Cimolesta (11 representatives) is a diverse order, thought to be ancestral or closely related to Ferae (e.g. [22]), the clade containing the extant orders Carnivora and Pholidota (pangolins), but have also been placed as a stem placental clade (e.g. [23]), as has Leptictida [24-26], of which there are three representatives in this dataset. Pantodonta (6 representatives) are sometimes considered to be related to Cimolesta (e.g. [27]), and are reconstructed by others as comprising an entirely separate order of placental mammal (e.g. [28]). Plesiadapiformes, of which there were two genera in this dataset, are often, but not uncontentiously, considered to be close to the origin of Primates [29]. Also included in this dataset were two palaeanodonts, a group which has been considered ancestral to pangolins [30], and two creodonts, which are often reconstructed as a paraphyletic group of stem carnivorans (e.g. [31-33]). By far the most troublesome polyphyletic grouping is that of “Condylarthra” (seventeen representatives), as well as “Acreodi” (five representatives), which have been commonly referred to as “archaic ungulates” [34]. Once thought to be ancestral to the now abandoned group “Ungulata” (a polyphyletic collection of extant hoofed mammals), these Palaeogene omnivores and herbivores have been suggested to be ancestral to several extant placental clades. For instance, phenacodontid “condylarths” have been affiliated with perissodactyls [35] as well as Afrotheria [36]. Arctocyonid “condylarths” such as *Chriacus* have been suggested to be ancestral to artiodactyls [37], with “Acreodi” often suggested as ancestral specifically to Cetacea [38]. Apheliscid “condylarths” have recently been suggested to be ancestral to Macroscelidea (elephant shrews or sengis), within Afrotheria [39,40].

Lastly within the eutherian sample were two genera of Zhelestidae, a small, herbivorous clade that has been placed as stem placentals, outside of the crown group, in a recent analysis [24], but have also been considered primitive “ungulatomorphs” by some (e.g. [41]).

Four unambiguous members of the placental stem were included (*Zalambdalestes*, *Bobolestes*, *Montanalestes*, and *Zhangolestes*), as was a single metatherian (*Asiatherium*) and two members of the therian stem lineage (*Arguimus* and *Kielantherium*), all of which are from the Cretaceous (145 to 66 Ma).

**Figure 1:**
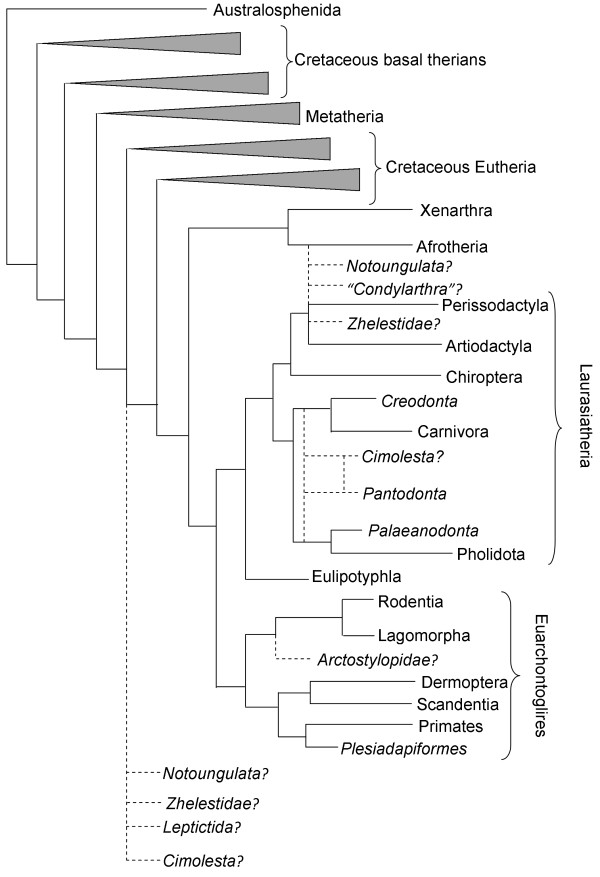
**High level phylogeny of mammals, including all groups used in this study. **Dotted lines represent possible affinities or where groups may be polyphyletic. Italicised taxonomic names are extinct groups, some of which are likely polyphyletic. Tree topology modified from Asher and Helgen [48], with extinct group placement based on various recent analyses or compilations [18,21-23,27,29,30,40,41]. This tree is intended to be illustrative of the diversity of groups covered in this analysis, and is not derived from any single phylogenetic analysis.

Finally, outside of Theria, a sample of Jurassic and Cretaceous australosphenidan mammals were included, comprised of two Cretaceous members of Monotremata (*Kollikodon* and *Steropodon*) and two members of the sister group Ausktribosphenida (*Asfaltomylos* and *Ausktribosphenos*). These extinct forms are generally considered to be closely related to modern monotremes (echidnas and the platypus) [15,42], although this has been disputed by some [43], and represent the final major division of crown mammalian diversity. Sampling, therefore, covers the majority of crown mammalian clades (see Figure 1).

All time periods from the Cretaceous to the Recent were well-represented in this sample. One taxon (*Asfaltomylos*) is known from the Middle Jurassic (174 to 163 Ma), 14 are known from the Cretaceous (145 to 66 Ma), 95 from the Palaeogene (66 to 23 Ma), and 25 from the Neogene (23 to 2.6 Ma). 9 taxa are extant. Twelve genera are known from both the Palaeogene and Neogene or from both the Neogene and Recent.

### Measurements

Measurements of lower molar length and width were obtained from the literature, either from published measurements or specimen images, or directly from museum specimens (both high-quality casts and original material) (Additional file 1). Area was estimated for each tooth as the product of length and width, following the method of both Polly [10] and Wilson [13]. For specimens that were measured directly, length and width were obtained from occlusal-view photographs in ImageJ v1.45s [44]. In addition, measurements for several taxa were obtained from the Palaeobiology Database (http://www.paleodb.org) on the 13^th^ of May 2012, using the taxonomic group name ‘Mammalia’ and the following parameters: Taxonomic resolution = ‘certainly identified to genus’, Body Parts = ‘m1, m2, m3’, with ‘all parts must be measured’ ticked. Output fields were ‘length’, ‘width’, ‘specimens measured’. All measurements were corrected for size by using the ratio of respective tooth area to that of m1 area, such that a posterior decrease in molar sizes would give values lower than 1, and an increase would give values larger than 1. Only specimens with two or all three adjacent molars present were included in the final dataset, and where multiple specimens were available, averages of molar ratios were analysed. All taxa which were composed solely of isolated molars, regardless of whether all three were represented, were removed from the dataset, due to inability to control for intraspecific variation. Ratios of m1:m2, m2:m3 and m1:m3 were quantified for each specimen, and averages of these ratios were then calculated for each genus. For taxa with more than three lower molars, only the first three were measured. Taxa with fewer than three lower molars were excluded from the analysis. Taxa for which either length or width were unavailable due to preservation were also excluded, such that length was not used as a proxy for area in any of the analyses.

While the approach used here, and in the studies noted above, estimated tooth area as a product of maximum length and width, some other studies [8,11,12,14] have measured tooth occlusal area directly. In order to establish the comparability of these area measurements, we also directly measured molar area in 41 genera (41 specimens) for which specimens were available. For this analysis, only values from the second lower molar were used, in order to reduce non-independence in the dataset, and molar area was measured from occlusal-view photographs using the outline tool in ImageJ v1.45s [44].

### Data analysis

#### Correlations among measurements of tooth size

For many fossil taxa, the nature of their preservation results in two-dimensional specimens, for which tooth widths (and hence areas) are unable to be assessed, except where preservation is in occlusal aspect. These specimens cannot be plotted in a tooth area ratio graph, although there is the potential for important size information to nonetheless be extracted. In order to identify whether molar length or width alone could be used as an accurate proxy for area, and hence increase the sample size in future studies, non-parametric Spearman Rank correlation analyses were performed among the relative lengths, widths and areas (scaled against the respective measure for m1) for each pair of molars. A strong length-area correlation would support the use of length as a proxy for molar area, and would imply that the major axis of increase in size is the antero-posterior axis. Such a result would further mean that the length ratios between teeth should follow the same pattern as area, although with differing regression parameters. All analyses were conducted in R [45].

#### Testing the inhibitory cascade model

**Figure 2:**
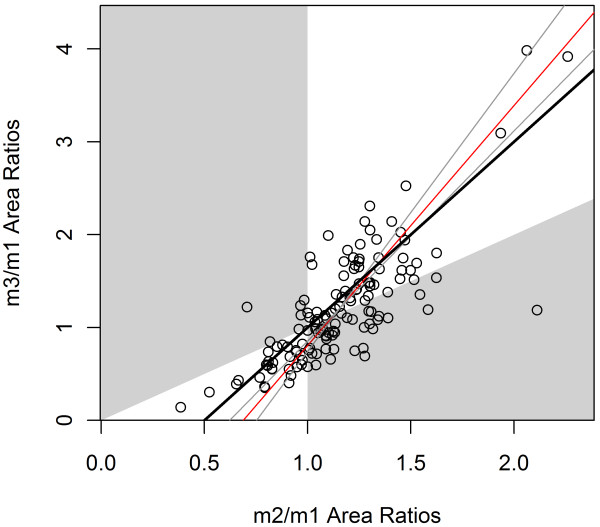
**Lower molar area ratios plotted for 132 mammalian genera, with regression line.** The black line represents the IC Model as predicted by Kavanagh *et al*. [8], with the white areas representing the predicted possible areas under the strict IC Model. Grey regions are outside the predicted areas of the model, and represent regions of the graph where m2 is either the largest lower molar (bottom-right) or the smallest (top-left). 65.2% of sampled taxa fall within the predicted area. The thin red line is the reduced major axis regression line, with 95% confidence bands in blue on either side.

Each taxon was plotted in a morphospace described by the ratios of molar areas of m2:m1 and m3:m1, as in previous studies (e.g. [8,10]), and a reduced major axis linear regression line was calculated (Figure 2). This regression line was then compared with the model predicted by Kavanagh *et al*. [8], as well as with the regression line of their original dataset, using 95% confidence intervals to test whether the two datasets were significantly different from one another or from the IC Model.

In order to test the second prediction of the IC Model – that m2 should occupy one third of total molar occlusal area – the proportion of total molar area occupied by m2 was calculated for all 154 specimens (132 genera) included in this study. Averages of this proportion were then taken for each genus. A two-tailed Student’s t-test was used to test whether the mean proportion of occlusal area taken up by the m2 was significantly different from 33%. This was then compared with the murine data ([8], supplementary information), to which the same method was applied.

#### Phylogeny and diet

Non-parametric MANOVA were conducted in R [45] using the ‘adonis’ command line in the ‘vegan’ package [46], in order to test for significant clustering of different dietary guilds and of taxonomic orders within the morphospace. The 101 taxa from higher-level groups with five or more representatives (with a pooled Creodonta-Carnivora group and a Primates-Plesiadapiformes group) were included in the analysis of phylogenetic clustering, comprising 10 groups in total. Phylogenetic group and dietary assignments are detailed in Additional file 2. Decisions on taxonomic grouping follow McKenna and Bell [27] where possible, and otherwise refer to the original descriptive literature for any given genus. Dietary information was extracted from the Paleobiology Database where possible, and otherwise directly from the original descriptive literature. Where dietary assignments were not available for a particular genus, family- or subfamily-level dietary estimates were used. Because precise diets can be difficult to discern in extinct organisms and are continually debated, broad categories (folivorous, carnivorous, omnivorous, insectivorous, frugivorous and durophagous) were used, which, despite some inevitable overlap, should be relatively accurate. Moreover, some inaccuracy in dietary assignations should not obscure a strong pattern with regard to morphospace position and diet, if one exists.

## Results

### Correlations of measurements

Significant positive correlations were found between all pairings of tooth length, width and product area, for ratios of m2 and m1, as well as of m3 and m1 (Table 1). Unsurprisingly, the strongest correlations were of length or width with area, reflecting the dependence of the area measurement on length and width, while correlations between length and width were markedly weaker.

**Table 1:** Correlations between size parameters in lower molars

**Correlation**	**Sample size**	**S-statistic**	**p-value**	**rho**
Length: Width, m2/m1	130	300091.2	<<0.001	0.4214
Length: Width, m3/m1	121	136839.2	<<0.001	0.6430
Length: Area, m2/m1	130	80543.3	<<0.001	0.8447
Length: Area, m3/m1	121	22257.7	<<0.001	0.9419
Width: Area, m2/m1	130	99563.7	<<0.001	0.8080
Width: Area, m3/m1	121	57711.7	<<0.001	0.8494

Pairwise Spearman Rank Correlation analysis between length, width and area values. Data used are ratios of m2/m1 and m3/m1 to control for absolute size differences.

The single strongest correlation observed was between length and area, particularly for m3:m1 ratios, suggesting that tooth length can reasonably be used as a proxy for tooth area. Spearman rank correlation analysis of the two different methods of measuring area, directly from specimen images or as the product of length and width, was highly significant (rho = 0.9878, p<<0.001), suggesting that product area is an accurate means of estimating tooth area.

### Comparison with IC model and previous studies

In the morphospace defined by m2/m1 against m3/m1, the majority of taxa (86 of 132) fell within the region predicted by the IC Model, although many specimens were found to be outside this region (Figure 2). This observation is consistent with, although slightly higher than, other studies, in which 12-20% fall outside the predicted region [10,12]. Of the 46 taxa which fell outside this ‘m2 intermediate’ region, 39 fell in the area in which m2 was the largest lower molar; only 7 displayed an m2 that was the smallest of the three molars. 30 genera exhibited molars that decreased in size posteriorly, and 56 exhibited molars increasing in size posteriorly. The 95% confidence intervals of the slope and intercept parameters (Table 2) overlapped with those of the murine study [8], but were significantly different from those of the arvicoline study [14]. The regression parameter confidence intervals of this study were, however, significantly different from the theoretical parameters of the IC Model (Table 2).

**Table 2:** Comparison of regression parameters in different analyses

**Model source**	**Slope**	**Slope 95% CI**	**Intercept**	**Intercept 95% CI**
IC Model	2.000	n/a	-1.000	n/a
Kavanagh *et al. *(2007) (murine rodents)	2.150	1.772:2.688	-1.219	-1.651:-0.925
Renvoisé *et al. *(2009) (arvicoline rodents)	1.390	1.208:1.555	-0.313	-0.407:-0.213
Asahara (2013) (Canidae)	0.450	0.376:0.515	-0.080	-0.104:-0.037
This Study (Mammalia)	2.303	2.007:2.655	-1.455	-1.863:-1.113

A comparison of the parameters of the IC Model, and linear regressions for three previous analyses of the IC Model, as well as this study.

Mean second lower molar area was 34.58%, similar to the 1/3 of total molar area predicted by the IC Model. However, both this study and the murine data give a value slightly higher and significantly different from the predicted value, although, as with the regression parameters, the two studies were not significantly different from one another (Table 3). In both cases, m2 comprised slightly more than one third of total molar occlusal area.

**Table 3:** m2 area as a proportion of total occlusal area

**Data source**	**t**	**df**	**m2 area as %**	**95% CI**	**p-value**
IC Model	n/a	n/a	33.33	n/a	1
Kavanagh *et al. *(2007) (murine rodents)	5.702	28	34.84	34.30:35.38	**<0.001**
This Study (Mammalia)	3.898	129	34.58	33.94:35.22	**<0.001**

A comparision between the proportion of total lower molar occlusal area (m1 + m2 + m3) taken up by m2, in the IC Model’s prediction, a previous analysis, and this study.

### Phylogeny and diet

**Figure 3:**
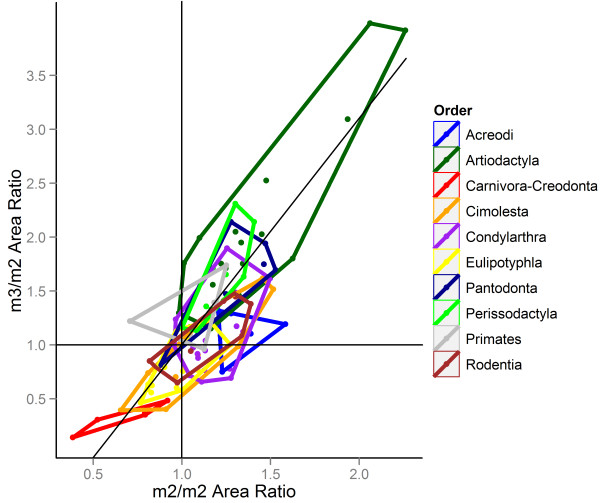
**Minimum area polygons for the ten taxonomic groupings. **Only groups with more than five genera were included in this analysis. Carnivora and Creodonta have been grouped together as possibly closely-related carnivorous placentals; Primates and Plesiadapiformes are also grouped together. Non-parametric MANOVA results in a highly significant clustering by taxonomic group (p<0.001), even when removing the most extreme members of Artiodactyla. The only group to overlap with the range of the carnivorous placental grouping is Cimolesta. “Condylarths” and Acreodi are clustered together in an area distinct from that occupied by Artiodactyla and Perissodactyla, with only three “condylarths” overlapping in range with the extant ungulate groups, showing that “archaic” and extant ungulates possess clearly distinct tooth morphologies.

**Figure 4:**
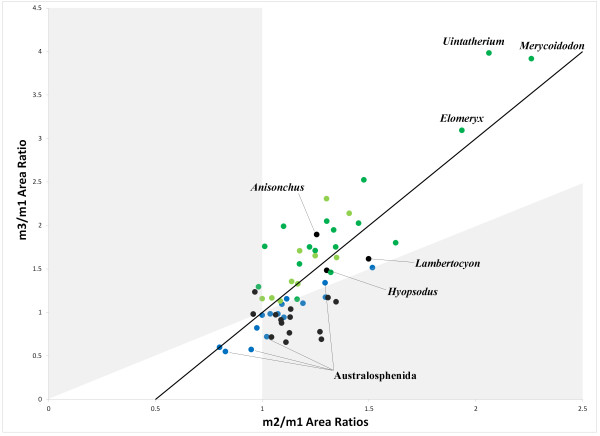
**Morphospace positions for Mesozoic mammals and the extant and “archaic” ungulates. **Mesozoic mammals (coloured in blue) are found near the centre (1,1) of the morphospace, closer to the plesiomorphic conditions of equal-sized molars. “Condylarths”, coloured black, are found mostly in the region of the morphospace where m2 is the largest molar, and are separate from Artiodactyla (dark green) and Perissodactyla (light green), with the exceptions of *Hyopsodus*, *Anisonchus* and *Lambertocyon*, all of which possess molars that increase in size posteriorly. Artiodactyla show the most extreme increase in molar size, with *Uintacyon*, *Elomeryx *and *Merycoidodon *exhibiting six-fold or more increases in molar area from m1 to m3.

Above the ordinal level, a non-parametric MANOVA indicated very strong clustering by taxonomic group (Table 4), with the Carnivora-Creodonta grouping particularly distinct from other taxonomic divisions (Figure 3). While clustering of orders was apparent in the dental morphospace, for example between rodents and primates – the two largest groups comprising Euarchontoglires – the relationships between these broad taxonomic groupings is not sufficiently well-resolved to identify any inter- or intraordinal patterns. Nonetheless, these results demonstrate that basal groups (australosphenidans, stem therians and stem placentals) consistently occupy the very centre of the morphospace (Figure 4), where tooth size is equal or subequal along the tooth row. The majority of the polyphyletic “condylarths” clustered together in the dental morphospace, but with the exception of the hyopsodontid *Hyopsodus*, arctocyonid *Lambertocyon* and periptychid *Anisonchus*, fell in a distinct region from the extant ungulate clades, Artiodactyla and Perissodactyla.

**Table 4:** Morphospace clustering due to diet and phylogeny

**Test**	**Df**	**Sum Sqs**	**Mean Sqs**	**F**	**R2**	**p-value**
Phylogeny	24	2.088	0.087	5.764	0.564	**<0.001**
Phylogeny (5+)	9	1.706	0.190	12.595	0.555	**<0.001**
Diet	5	0.922	0.184	8.351	0.248	**<0.001**
Diet (reduced)	5	0.736	0.147	7.768	0.243	**<0.001**

Results of non-parametric MANOVA testing clustering due to phylogeny and diet in the morphospace. All results were highly significant. Sample sizes were 132 genera for of Phylogeny and Diet, 101 for the limited phylogenetic groupings (5+ representatives per group), and 127 for the reduced dietary analysis (without extreme outliers, of which four were artiodactyls and one an arctostylopid).

**Figure 5:**
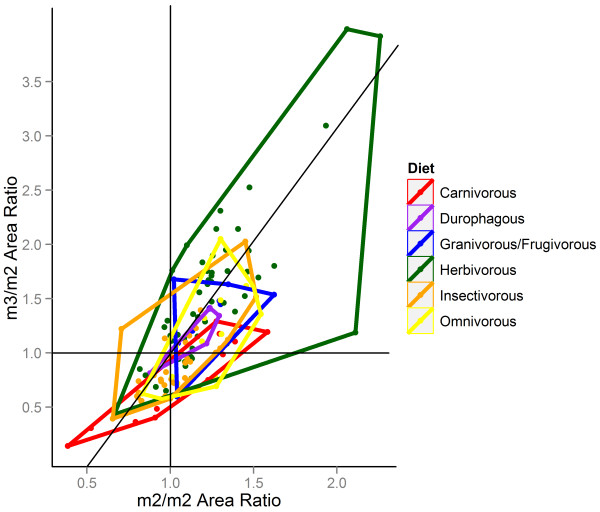
**Minimum area polygons for 132 genera divided into six dietary types. **The data show a similarity to the predicted distribution from Kavanagh *et al.*[8], with more faunivorous dietary types placed in the bottom left of the morphospace, and more herbivorous dietary types in the top right.

Dietary groups were was also found to cluster significantly in molar morphospace (Table 4, Figure 5). Such analyses are often susceptible to outliers, and so the five outlying and likely herbivorous taxa (the artiodactyls *Uintatherium*, *Merychyus*, *Merycoidodon*, and *Elomeryx*, and the arctostylopid *Palaeostylops*) were excluded from a second, otherwise identical analysis. While the F-statistic was lowered, the degree of clustering remained highly significant, suggesting that there is a robust relationship between relative tooth areas and diet.

## Discussion

The results of this study are consistent with the hypothesis that the IC Model of lower molar development is the plesiomorphic condition for Mammalia. Furthermore, this study is wholly consistent with, and resembles closely, the results from a recent study that focused on rodents [12]. While murids made up the majority of the rodents in this study, Labonne *et al.*[12] used a broader phylogenetic sampling of rodent taxa, which spanned the same range of molar ratios as do all mammals. This correspondence strongly suggests that a common developmental mechanism underlies the development of all mammalian teeth, rather than being specific to rodents.

Deviations from the parameters of the IC Model have been identified in a few clades – such as arvicoline rodents [14] and canids [11], both of which show a significantly lower slope than predicted by the IC Model. This latter group’s deviation from the model has been hypothesised to relate to the presence of the specialised m1, which forms part of the carnassial pair of slicing teeth. In each case, however, the observed data fell within the region of morphospace consistent with the predictions of an inhibitory cascade, even if the parameters of the regression line differed. High variability has also been noted in South American ungulates [13], with two groups (Astrapotheria and Interatheriidae) deviating significantly from the IC Model, as well as falling outside of the predicted region of morphospace.

In the IC Model, the value of the slope is determined by the degree to which the effect of the activator/inhibitor mechanism is changed in the m3:m1 ratio with respect to the m2:m1 ratio. It can therefore be described as representing the change in effect of the inhibitory cascade mechanism along the tooth row. In the pure IC Model, the expected value is 2, meaning that the change is cumulative and additive; m3 has had twice the effect of the activator/inhibitor balance as has m2. The difference in gradient of the slope, then, if expressed in terms of the change of effect through the molar series, would suggest that, through evolutionary time, the degree to which the effect changes along the tooth row is easily modified, and the morphologies of murine rodents, arvicoline rodents and canids may be explained through relatively small changes in this balance.

That 65% of sampled taxa in this study fell within the area predicted by the model suggests that the IC Model is indeed a common pattern underlying mammalian molar development. This percentage rises to 75% of taxa if excluding the 22 ‘archaic ungulates’, 18 of which fell in the m1<m2>m3 region of the morphospace. These ‘condylarths’ are considered by most to represent a polyphyletic group (e.g. [41]), so their close proximity to one another, and distinct position in the morphospace from the extant ungulate clades, Artiodactyla and Perissodactyla, is notable. This observed separation between ‘archaic’ and extant ungulates suggests that these taxa may utilise distinct molar developmental mechanisms, with the ‘archaic’ ungulates representing a deviation from the common mammalian pattern. While this result is not necessarily surprising, as ‘condylarths’ are not as a whole considered to represent the ancestral group for modern ungulates, the observed tight clustering of the sampled condylarths, along with the sampled representatives of Acreodi (*Eoconodon, Ankalagon, Oxyclaenus, Sinonyx* and *Mesonyx*), is surprising. It is, however, plausible that the clustering of the ‘Condylarthra’ reflects their shared omnivorous to herbivorous dietary condition, rather than phylogenetic proximity, since dietary groups were also strongly clustered within the molar morphospace. A well-supported tree is required to analyse the real association between phylogeny, diet and dental morphospace proximity. Ongoing work to resolve the position of Palaeocene condylarths within the broader placental mammal tree should ultimately allow for a more robust test of phylogenetic clustering within this dataset, and provide insight into whether this grouping represents a taxonomic or ecological signal, if either.

Apparent differences from the IC Model in previous studies may be largely due to limited taxonomic focus. For example, our data show that both arvicolines [14] and murines [8], while distinct in relative molar size from one another, fall within the range of observed variation for mammals as a whole, as well as within the region of morphospace that the IC Model predicts. While variation in the regression parameters of subgroups is high [13], the IC Model is generally consistent with the higher-level pattern observed across Mammalia. That the second prediction of the IC Model is not upheld, with the second molar being slightly, albeit significantly larger than would be expected, is not unsurprising given that the majority of taxa which fall outside of the predicted area do so with m2 as the largest tooth. Again, the data are consistent with the murine data collected by Kavanagh *et al.*[8], which indicates further that, while some deviations are apparent, the patterns observed for the majority of mammalian subgroups are consistent with that for Mammalia as a whole.

Despite the significant clustering of dietary groups, molar ratios likely do not provide a useful predictive tool for estimating diet in extinct organisms, as there is extensive overlap of several dietary groups in the central region of the molar morphospace (Figure 5). However, if molar proportions fall in the more extreme regions of the space (i.e., when m1>>m2>>m3 or vice versa), a prediction of herbivory (where m3 is largest) or carnivory (where m3 is smallest) could be made with reasonable confidence. Additional complications arise from the inherent association between phylogeny and diet, especially as some extinct genera in this study were assigned diets based on those of con-familial genera where more specific data were unavailable. An explicitly phylogenetic analysis, which will only be possible once a resolved phylogenetic tree is available, as well as better understanding of diet in many of these taxa, would greatly improve the ability to distinguish these two effects.

Another interesting aspect of molar development that is not considered here concerns the role of the premolars. Labonne *et al.*[12] demonstrated that loss of the lower fourth premolar in some taxa appeared to remove a spatial constraint on the development of the lower first molar. This loss would then affect the development of the first molar to a far greater extent that the more posterior molars, enabling a proportionally larger m1. None of the taxa included in this study are known to lack a fourth premolar, with the exception of the single metatherian genus, which, like all metatherians, has only three premolars. The effect should not influence the results presented here, but is important for future studies to take into account.

## Conclusion

In conclusion, the results presented here corroborate the hypothesis that the Inhibitory Cascade Model is plesiomorphic to Mammalia as a whole. Although exceptions do exist, including many ‘condylarths’ these are more likely to represent secondarily derived states, rather than alternative ancestral conditions for the broader clade, as nearly all basal Mammaliaforms fall within the predicted area for the model. That the IC Model applies to mammalian taxa ranging from Jurassic and Cretaceous australosphenidans (*Asfaltomylos*, *Ausktribosphenos*, *Kollikodon*, and *Steropodon*) to early Cretaceous stem therians (*Arguimus*, *Bobolestes*, and *Kielantherium*), to a diverse sample of Cretaceous to Recent eutherians, including crown placentals, suggests that this developmental constraint predates the divergence of Australosphenida and Boreosphenida (marsupial and placental mammals and their stem groups) approximately 180 Ma [15,47]. As many of the most basal taxa in this analysis fall near the centre of the molar morphospace, where all three lower molars are near-equal in area, one could further hypothesise that a trend along the predicted regression line towards either a larger m1 or a larger m3 corresponds with dietary specialisation through mammalian evolution. Better resolved phylogenetic trees of living and extinct mammals are required to further reconstruct the trajectory of molar size evolution across Mammalia, but further work on the distribution of these changes, as well as the effect of different ecological parameters, will provide important new information and models to reconstruct the evolution of mammalian dental morphology and diversity both today and in the fossil record.

## Availability of supporting data

The data set supporting the results of this article is included within the article and its additional file.

## Abbreviations

IC: Inhibitory cascade; Ma: Million years ago; m1: First lower molar; m2: Second lower molar; m3: Third lower molar.

## Competing interests

The authors declare that they have no competing interests, financial or otherwise.

## Authors’ contributions

TJDH collected data, performed analyses and wrote the manuscript. AG conceived and designed the study and wrote the manuscript. Both authors read and approved the final manuscript.

## Supplementary Material

Additional file 1 **Tooth measurement values. **Measurements of lengths and widths of lower molars in 192 specimens of mammal, calculations of area and ratios, and details of the source of the data. Click here for file

Additional file 2 **Taxonomic and dietary assignments. **Dietary guild, higher taxonomic level and area ratios for all 132 observed genera. Click here for file
